# Total Knee Arthroplasty After Ipsilateral Below-knee Amputation: A Review of the Literature and Surgical Techniques

**DOI:** 10.1016/j.artd.2022.03.020

**Published:** 2022-06-17

**Authors:** Katherine Dong, Anna Cohen-Rosenblum, Molly Hartzler

**Affiliations:** a Department of Orthopaedic Surgery, Louisiana State University Health Sciences Center, New Orleans, LA, USA; b Dignity Health Medical Foundation, Redding, CA, USA

**Keywords:** Osteoarthritis, Total knee arthroplasty, Below knee amputation, Prosthetics, Rehabilitation

## Abstract

Patients with knee osteoarthritis in the setting of ipsilateral below-knee amputation present a challenge in terms of patient positioning, intraoperative assistance, implant alignment, postoperative rehabilitation, and prosthesis adjustment. This is a report of a patient with a history of below-knee amputation with ipsilateral knee pain due to osteoarthritis, treated with elective total knee arthroplasty. This was done using custom cutting blocks made via preoperative computed tomography scans, and a single assistant as well as a large hip bump and lateral support were used for positioning. The patient was weight-bearing as tolerated in his regular below-knee prosthesis starting from postoperative day 1, with 1 prosthetic adjustment made during the first week of rehabilitation. The patient was pain-free with full range of motion at 1-year follow-up.

## Introduction

Osteoarthritis is the most common joint disorder in the United States, with symptomatic knee osteoarthritis affecting about 10%-13% of the population older than 60 years [1]. End-stage knee osteoarthritis is typically treated with total knee arthroplasty (TKA) after conservative measures have failed. The current incidence of TKA in the United States is over 670,000 knees per year [2]. Lower limb amputation is also a common procedure with 150,000 lower limb amputations performed annually in the United States [3]. Ipsilateral below-knee amputation (BKA) is not a contraindication to TKA; however, these cases are rare with few case reports in the literature and present an additional set of considerations for the surgeon.

Patients with knee osteoarthritis in the setting of ipsilateral BKA present an interesting challenge for the orthopedic surgeon. Patient positioning, intraoperative assistance, implant alignment, postoperative rehabilitation, and prosthesis adjustment are all important considerations. We present a case of TKA in the setting of ipsilateral BKA, as well as a literature review and clinical tips for surgeons who may encounter this situation.

## Case history

**Figure 1:**
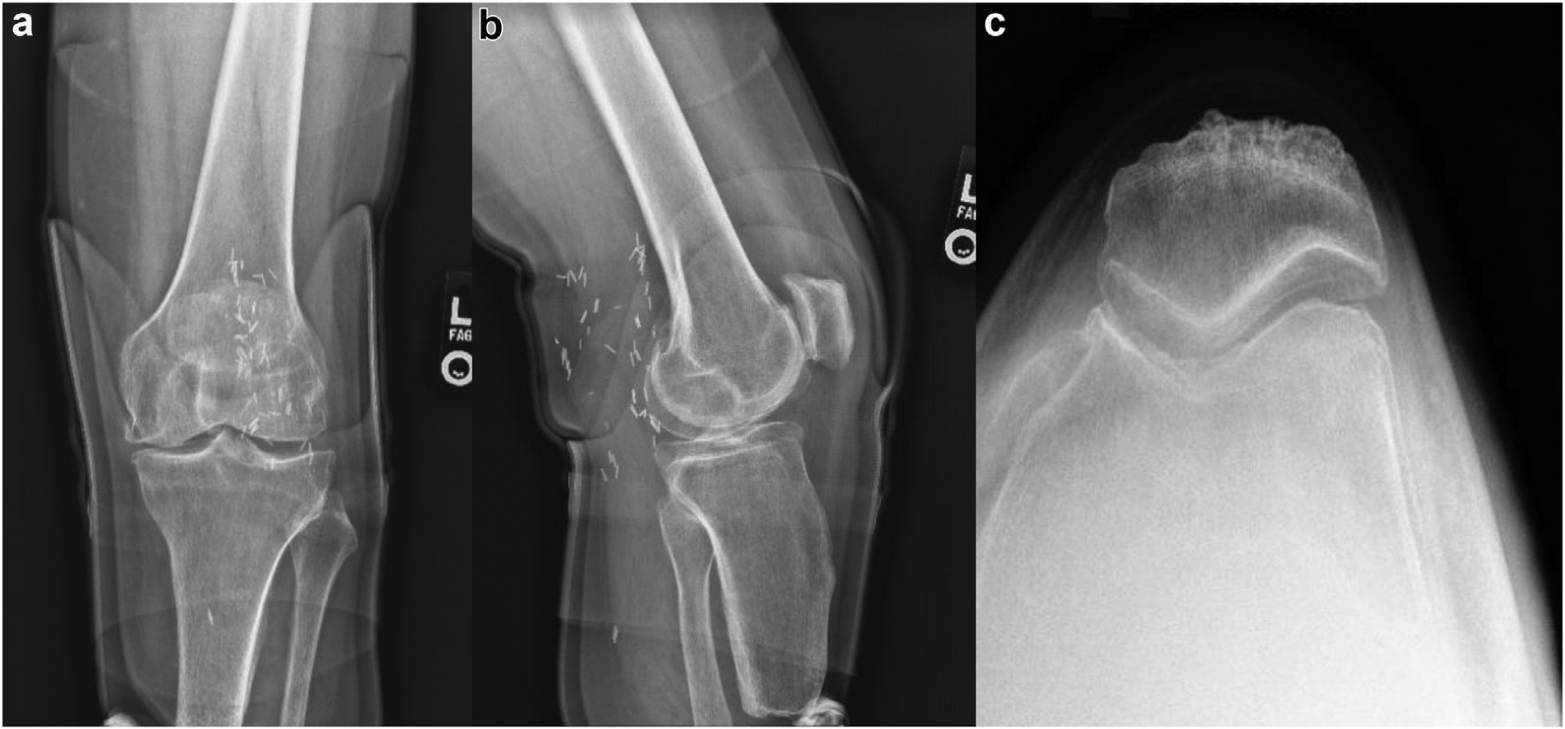
Preoperative radiographic imaging of the right knee: (a) anteroposterior, (b) lateral, and (c) sunrise view.

Informed consent was obtained from the patient to use their demographic and case history for publication. This patient is a 52-year-old gentleman with a history of peripheral artery disease and a body mass index of 31 kg/m^2^ who underwent left BKA after failed revascularization of the left foot. He then underwent revision amputation approximately 6 years prior to presentation due to wound healing issues and distal tibial osteomyelitis; ultimately, the infection was eradicated. He underwent a right (contralateral) TKA in 2018 for knee pain due to osteoarthritis that was uncomplicated. He presented with tricompartmental arthritis of the left knee and a well-healed distal residual limb (Fig. 1). On exam, he had tenderness to palpation about the left knee medial and lateral joint line. Range of motion was approximately 0°-135° of flexion, with pain and crepitus throughout the range of motion. He had a mild effusion. He had no pain with passive range of motion of the left hip and a negative Stinchfield test. The left lower extremity was warm and well perfused, with a palpable popliteal pulse. He had a well-fitted left BKA prosthesis and was active.

The patient had been through a full complement of conservative treatments including physical therapy, nonsteroidal anti-inflammatory medications, and intraarticular steroid injections, and after a discussion of the risks, benefits, and alternatives, we elected to proceed with a left TKA. Preoperative risk evaluation was obtained from the patient’s primary care physician and cardiologist, and inflammatory markers were not elevated, so no further preoperative infection workup was done.

**Figure 2:**
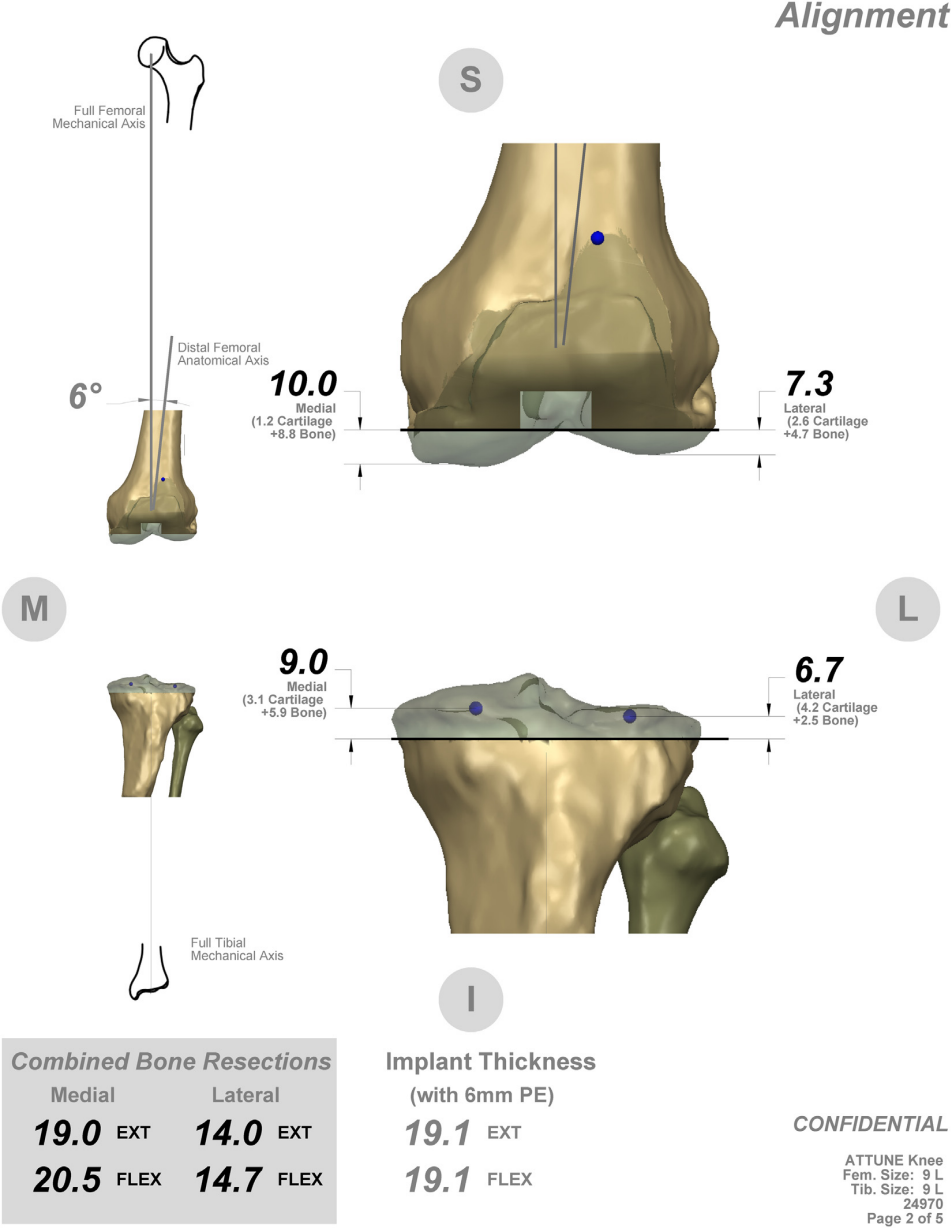
Preoperative template of custom cutting blocks.

Given the patient's abnormal anatomy and history of infection, preoperative planning was achieved through computed tomography scan with the patient's prosthesis on, and custom TRUMATCH (Depuy Synthes, Warsaw, IN) cutting blocks were fabricated for the Attune (Depuy Synthes, Warsaw, IN) cemented posterior stabilized TKA (Fig. 2). In this way, the amount of bone resection to achieve neutral mechanical alignment with the operative lower extremity was determined without needing to instrument the tibial canal.

**Figure 3:**
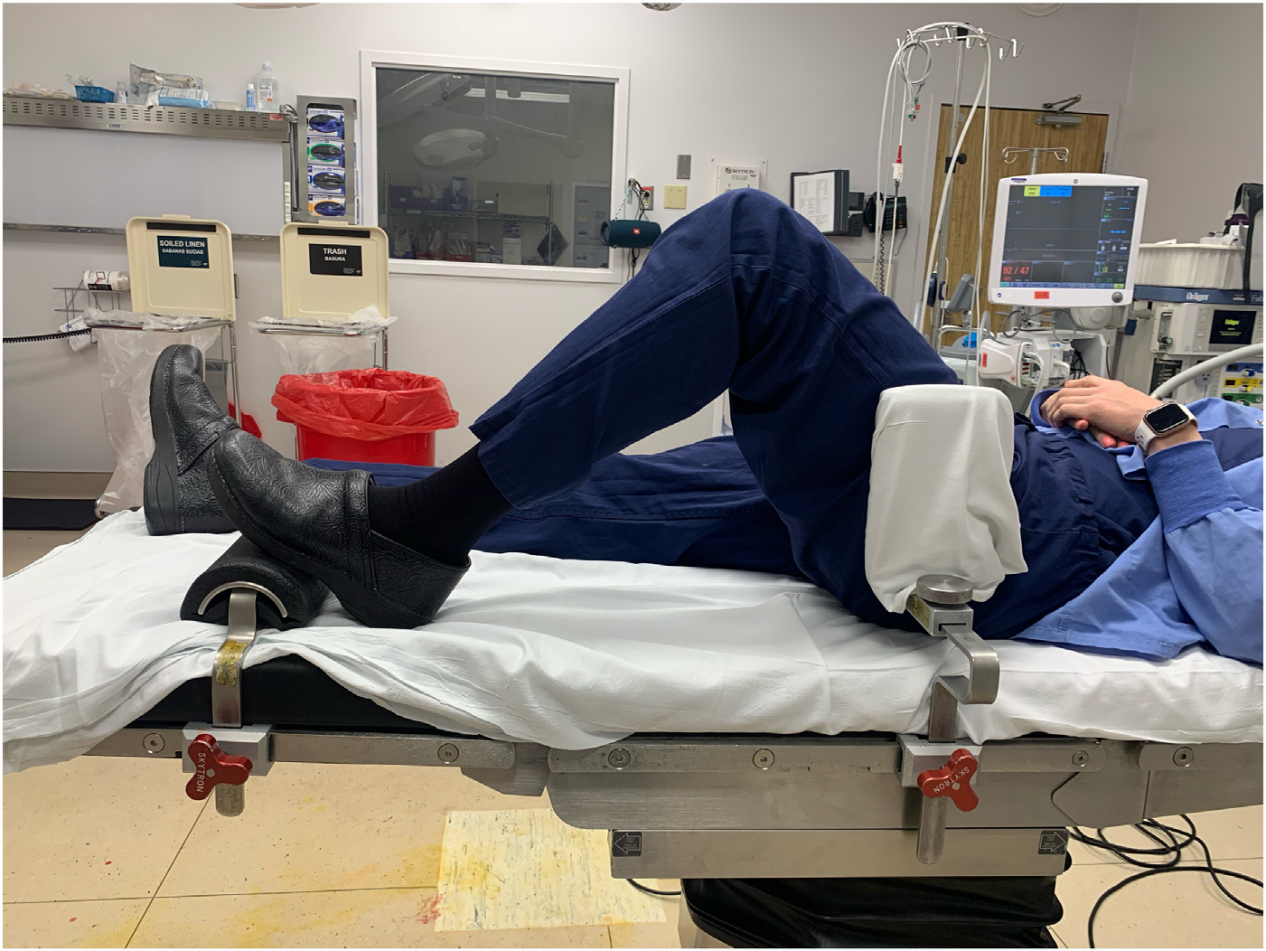
KneeGRIP (Sunmedica, Redding, CA) positioning system enabling procedure to be performed with a single assistant.

Intraoperatively, the patient's thigh was supported by a large bump and lateral upright support from the kneeGRIP (Sunmedica, Redding, CA) system (Fig. 3). A single assistant was present for the case. A midline skin incision cheating slightly medial to avoid the thinner skin over the tibial tubercle and a medial parapatellar arthrotomy were used. Tibial exposure was facilitated using a Mikhail retractor (Johnson & Johnson, New Brunswick, NJ) placed posterior to the tibia after standard medial tibial release and take-down of the anterior and posterior cruciate ligaments, allowing full tibial subluxation. Full tibial subluxation was helpful for seating the tibial custom cutting block without disrupting the bony anatomy. Gap balancing technique was used with a tensioner. Due to the patient's soft bone quality, stems were placed in the tibial and femoral components, and ultimately a varus-valgus constrained polyethylene insert (Depuy Attune CRS tibial insert; Depuy Synthes, Warsaw, IN) was utilized for slight lateral laxity. Due to the patient’s history of peripheral vascular disease, the tourniquet was only inflated during cementation of the components. Due to the patient’s history of distal tibial osteomyelitis, antibiotic cement was used, and instrumentation of the tibial canal was avoided. The skin incision was closed with running 3-0 barbed monofilament suture over 3-0 interrupted vicryl suture and skin adhesive and covered with an occlusive silver-impregnated dressing.

Postoperatively, the left lower extremity was placed in a compression wrap, and he was transitioned to his regular sock and silicone stump sleeve on postoperative day 1. He began to weight-bear as tolerated from postoperative day 1 with daily surveillance of the incision. This decision was made as the patient had already completed the rehabilitation process for a TKA on his contralateral lower extremity and was concerned about not achieving optimal outcomes if he were not able to immediately ambulate and fully participate with physical therapy. The risk of postoperative wound complications with immediate weight-bearing (as opposed to waiting for postoperative edema to resolve before ambulating with his prosthesis) was explained, and the patient agreed to very close follow-up of his incision.

**Figure 4:**
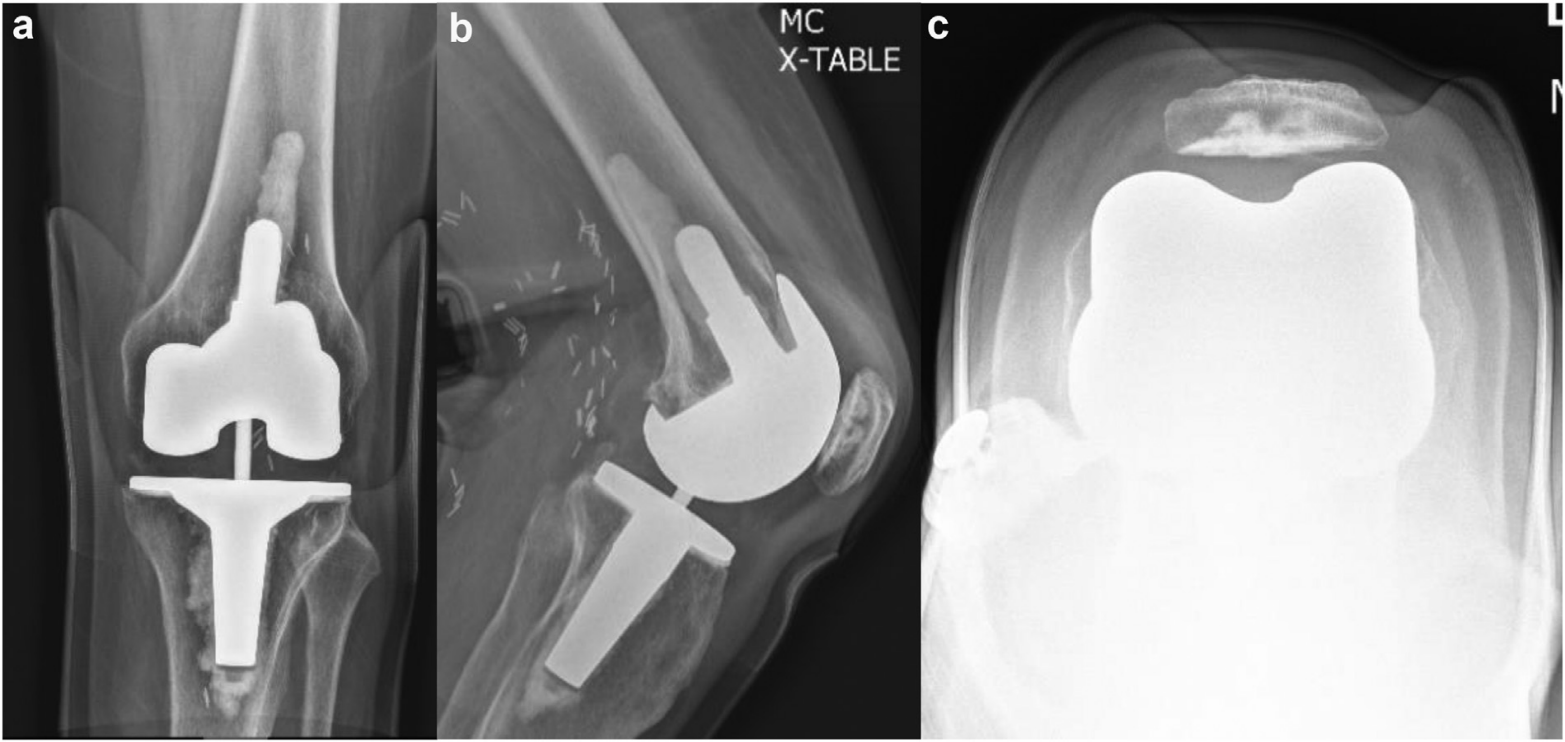
Postoperative radiographic imaging of the right knee: (a) anteroposterior, (b) lateral, (c) sunrise view.

He had 1 adjustment made to his prosthesis during the first week after surgery. At 2 weeks, he had minimal swelling, his incision was healing without issue, and his range of motion was 5°-115°. At 6 weeks, his incision was well healed and range of motion was 0°-130°. At 3-month and 1-year postoperative follow-up, his range of motion remained full, radiographs showed no signs of loosening and appropriate alignment (Fig. 4), and he was pain-free and had returned to all desired activities.

## Discussion

Ipsilateral knee replacement of below-knee amputees is extremely uncommon, with most of the literature describing degenerative changes necessitating TKA on the contralateral side [[4], [5], [6], [7], [8], [9], [10], [11]]. This is likely due to stresses on the contralateral limb from altered gait mechanics with a prosthesis [12]. Osteoporosis and disuse atrophy after BKA may in fact decrease the incidence of ipsilateral osteoarthritis [6]. If the ipsilateral knee develops symptomatic osteoarthritis after BKA, the prosthesis can be adjusted to offload the affected compartment in addition to traditional conservative management [13].

If conservative management fails, consideration should also be given to procedures other than TKA, including osteotomy, above-knee amputation, or arthrodesis. Osteotomy could correct a periarticular deformity and offload painful areas of the knee and has been described, for example, in a case of a proximal tibial malunion after BKA causing a bony prominence and skin breakdown [14]. However, realignment osteotomy is generally contraindicated in cases of symptomatic arthritis in more than 1 compartment, and an osteotomy incision would potentially create wound-healing difficulties should the patient ever need to be converted to a TKA and/or need staged procedures for removal of hardware followed by TKA [15]. Above-knee amputation would essentially remove the pain generator of the osteoarthritis by removing the knee joint itself but requires increased energy expenditure during ambulation compared with a BKA [16]. Knee fusion would also remove the pain generator by preventing any motion at the knee but affects the biomechanics of ambulation with adverse consequences for nearby joints [17].

In this case, the patient's symptomatic tricompartmental arthritis and failure of conservative management with physical therapy, nonsteroidal anti-inflammatory medications, and intraarticular steroid injections led to the recommendation of surgical treatment with TKA. Based on the experience with this case as well as a review of the existing literature, the main challenges of TKA after ipsilateral BKA include surgical positioning, tibial component alignment, and postoperative rehab protocol [[8], [13], [18], [19], [20], [21], [22], [23], [24]]. A brief overview of studies describing BKA after ipsilateral TKA, including 8 patients with 9 TKAs, is given in Table 1.

**Table 1:** Review of BKA after TKA literature.

Paper	Positioning	Assistants	Tibial alignment	Implant	Prosthesis fitting	Complications	Follow-up
Pasquina, 2000 [13]	n/a	n/a	n/a	Cement	POD 4 temporary prosthesis, 6 wk final	POD 28 peri-incisional cellulitis treated with PO abx	1 y
Crawford, 2003 [18]	Box covered with sterile drapes	2	Intramedullary	CR, cement	6 wk	None	8 mo
Vanin, 2008 [19]	n/a	n/a	Extramedullary	CR, cement	Immediate temporary prosthesis	None	2 y
Konstantakos, 2008 [20]	Sterilized plaster mold of prosthesis	n/a	Extramedullary	PS, cement	3 wk	None	8 y
Dudhniwala, 2011 [21]	Side support for thigh and distal “roll on a pole”	1	Intramedullary	PS with femoral and tibial stems, tibial metaphyseal sleeve (L), cement	6 wk	none	1 y (L)/7 y (R)
Amanatullah, 2014 [8]	n/a	n/a	n/a	PS, cement	n/a	None	2 y
Fleming, 2016 [22]	Bolster under thigh	2	Patient-specific cutting guide (preop CT)	PS, cement	n/a	None	4 mo
Putnis, 2020 [23]	Sling during prep, wedge under knee	2	Computer navigation	PS, cement	8 wk	None	1 y
Maupin, 2019 [24] (revision TKA w/ipsilateral BKA technique)	Hip bump, distal transverse foot bump, mid-thigh side support sterile radiolucent triangle	1	Intramedullary	n/a	n/a	n/a	n/a

CR, cruciate retaining; CT, computed tomography; POD, postoperative day; PS, posterior stabilized.

Positioning during BKA after TKA can be challenging, and a variety of techniques have been described, including a sterile box under the distal residual limb and 2 assistants [18], a sterilized plaster mold of the preoperative BKA prosthesis [20], a side-support for the thigh and distal “roll on a pole” support for the stump during flexion with a single assistant [21], a cylinder bolster under the thigh was used for stabilization during knee flexion [22], a wedge under the knee with 2 assistants [23], as well as a hip bump, distal transverse foot bump, mid-thigh side support to prevent external rotation of the hip, and a sterile radiolucent triangle with the stump being secured to the triangle with adhesive wrap [24]. In the case described here, adequate limb stability was facilitated with a large sterile bump under the thigh, the lateral upright positioner from the kneeGRIP (Sunmedica, Redding, CA) system, and a single assistant. Tibial exposure was achieved via subluxation of the tibia using a retractor posterior to the tibia, allowing for seating of the custom cutting guide.

Implant alignment is difficult due to lack of tibial anatomic landmarks and can be achieved with intramedullary guides [18,21], intraoperative navigation [23], patient-specific cutting guides [22], extramedullary guide estimating the position of the ankle via the proximal tibia [19], or fashioning a sterile distal prosthesis to use for reference [20]. We selected custom cutting guides based on a computed tomography scan done with the patient's prosthesis in place to allow the patient's prosthetic ankle center to guide the tibial cut, thereby avoiding instrumenting a tibia with a history of osteomyelitis.

Stems were utilized secondary to soft bone, which is common in the distal residual limb of amputees [25]. Tibial and femoral stems have been shown in biomechanical studies to decrease compressive stress on the surrounding bone, making them an attractive option in cases of osteoporosis, bone loss, or obesity [[26], [27], [28], [29]]. In addition, in cases of using varus-valgus constraint, the addition of a tibial stem extension is thought to be beneficial in transferring the increased stress generated by the constraint to a larger surface area, which in turn could lead to decreased risk of future loosening [30].

Rehabilitation typically consists of non-weight-bearing until resolution of postoperative edema and fitting of a new prosthesis. This non-weight-bearing period in the literature has ranged from 4 days to 8 weeks, with no conclusive recommendation [[13], [18], [19], [20], [21], [23]]. Postoperatively, our patient was allowed to weight bear as tolerated in his prosthesis with close surveillance of the incision. This facilitated early physical therapy without significant modification to the program. He healed without any wound complications and achieved full motion and resolution of preoperative pain.

## Summary

This report describes a patient with osteoarthritis of the knee and a history of ipsilateral BKA and tibial osteomyelitis who underwent a successful TKA. These patients comprise a unique population with special considerations for surgical positioning, tibial component alignment, and postoperative rehab protocol but generally have good clinical results.

**Key Points**

• Consider using custom cutting guides if the patient has minimal distal residual limb or a history of infection in the distal limb.
• A second assistant may be useful depending on the body habitus of the patient and preoperative range of motion but may not be necessary with appropriate positioning equipment.
• Full subluxation of the proximal tibia is helpful for placement of the tibial cutting guide.
• Careful soft-tissue handling and skin closure are important as these patients may have a history of wound healing problems and/or peripheral vascular disease.
• The decision to allow early weight-bearing should be individualized depending on risk factors for delayed wound healing, but this case demonstrates that weight-bearing in the patient's regular prosthesis can be safely resumed on postoperative day 1, allowing for early mobility and physical therapy.

## Conflicts of interest

The authors declare the following financial interests/personal relationships which may be considered as potential competing interests: A. C.-Rosenblum receives financial or material support from JBJS and Elsevier; is in the editorial or governing board of Arthroplasty Today and Journal of Arthroplasty; and is a board member of AAHKS Young Arthroplasty Group, AAHKS Nominating Committee, and RJOS Education Committee. M. Hartzler is in the speakers' bureau of or gave paid presentations for DePuy Synthes.

For full disclosure statements refer to https://doi.org/10.1016/j.artd.2022.03.020.

## Informed patient consent

Informed consent was obtained from the patient to use their demographic and case history information for this case report.

## Appendix ASupplementary data

Conflict of Interest Statement for Cohen-Rosenblum

Conflict of Interest Statement for Dong

Conflict of Interest Statement for Hartzler
