# Supplementary material for: Total Knee Arthroplasty After Ipsilateral Below-knee Amputation: A Review of the Literature and Surgical Techniques
Source: Arthroplast Today. 2022 Jun 17;16:158–63. doi: 10.1016/j.artd.2022.03.020 (PMC9234005; doi:10.1016/j.artd.2022.03.020)
Supplement: Conflict of Interest Statement for Cohen-Rosenblum [file mmc1.pdf]

CONFLICT OF INTEREST STATEMENT

American Association of Hip and Knee Surgeons

(Adopted from the American Academy of Orthopaedic Surgeons disclosure statement)

The following form **must be filled out completely and submitted by each author (example, 6 authors, 6 forms). All items require a response. If there is no relevant disclosure for a given item, enter "*None*."**

Total knee arthroplasty after ipsilateral below knee amputation: A case report and review of the literature

Manuscript Title

1. Royalties from a company or supplier (The following conflicts were disclosed)
None

2. Speakers bureau/paid presentations for a company or supplier (The following conflicts were disclosed)
none

3A. Paid employee for a company or supplier (The following conflicts were disclosed)
none

3B. Paid consultant for a company or supplier (The following conflicts were disclosed)
none

3C. Unpaid consultants for a company or supplier (The following conflicts were disclosed)
none

4. Stock or stock options in a company or supplier (The following conflicts were disclosed)
none

5. Research support from a company or supplier as a Principal Investigator (The following conflicts were disclosed)
none

6. Other financial or material support from a company or supplier (The following conflicts were disclosed)
none

7. Royalties, financial or material support from publishers (The following conflicts were disclosed)
JBJS, Elsevier

8. Medical/Orthopaedic publications editorial/governing board (The following conflicts were disclosed)
Arthroplasty Today
Journal of Arthroplasty

9. Board member/committee appointments for a society (The following conflicts were disclosed)
AAHKS Young Arthroplasty Group
AAHKS Nominating Committee
RJOS Education Committee

Each author must sign AND print or type his/her name, date and submit a separate form

In addition, one BLINDED Conflict of Interest form (no author names used) should be submitted per manuscript with all author disclosures.

Anna Cohen-Rosenblum	[signature]	1/2/21
Author Name (Print or Type)	Author Signature	Date
